# Improvement in the Thermal Conductivity of Silver Epoxy Adhesive by Treating with Water Vapor

**DOI:** 10.3390/polym15102338

**Published:** 2023-05-17

**Authors:** Yiyang E, Zhaobo Tian, Keyu Chi, Renyao Jiang, You Lv, Qi Sun, Yuan Zhu

**Affiliations:** 1 School of Microelectronics, Southern University of Science and Technology, Shenzhen 518055, China; 2 Baopeng New Materials Technology Co., Ltd., Shenzhen 518055, China; 3 Southern Institute of Industrial Technology, Shenzhen 518055, China; 4 Foshan (Southern China) Institute for New Materials, Foshan 528000, China

**Keywords:** vapor, silver epoxy adhesive, thermal conductivity, electrical conductivity, silver particles

## Abstract

With the miniaturization of electronic devices, electronic packaging has become increasingly precise and complex, which presents a significant challenge in terms of heat dissipation. Electrically conductive adhesives (ECAs), particularly silver epoxy adhesives, have emerged as a new type of electronic packaging material, thanks to their high conductivity and stable contact resistance. However, while there has been extensive research on silver epoxy adhesives, little attention has been paid to improving their thermal conductivity, which is a critical requirement in the ECA industry. In this paper, we propose a straightforward method for treating silver epoxy adhesive with water vapor, resulting in a remarkable improvement in thermal conductivity to 9.1 W/(m·K), three times higher than the sample cured using traditional methods (2.7 W/(m·K)). Through research and analysis, the study demonstrates that the introduction of H_2_O into the gaps and holes of the silver epoxy adhesive increases the path of electron conduction, thereby improving thermal conductivity. Furthermore, this method has the potential to significantly improve the performance of packaging materials and meet the needs of high-performance ECAs.

## 1. Introduction

With the miniaturization of electronic equipment, electronic packaging has become increasingly complex and precise [1,2,3,4]. The use of electrically conductive adhesives (ECAs), which are thermoset polymers filled with metal fillers, is rapidly expanding due to their low cost and processing temperature [3,5]. ECAs are extensively used in the assembly and packaging of electronic devices such as high-density multilayer interconnect substrates and high-speed, high-frequency circuits [1,6,7,8,9,10]. Among all of the ECAs, silver epoxy adhesives are most commonly used because of their high conductivity and stable contact resistance [11,12,13,14].

In recent years, there has been a growing interest in the development of silver epoxy adhesives with improved electrical conductivity [15,16,17]. The electrical conductivity of these conductive compositions can be modified by selecting the proper size, shape, and type of filler [18,19,20]. However, few studies have focused on the thermal conductivity of these adhesives, which is a critical requirement for ECA industries, to address the heat dissipation problems generated by highly integrated circuits. For instance, in the study conducted by Cui [21], an 85 wt% silver powder content was used to prepare a silver epoxy adhesive. However, the resulting thermal conductivity in the vertical direction was found to be only 5 W/(m·K) which is insufficient for practical applications. Therefore, the development of more thermal conduction pathways is expected to further improve the thermal conductivity of silver epoxy adhesives.

Taking into account the ability of vapor to penetrate gaps and establish channels for the transfer of thermal and electrical energy, we devised a simple method for enhancing the thermal and electrical conductivity of silver epoxy adhesive by treating it with water vapor. Additionally, we analyzed the mechanism of vapor penetration, and improved the thermal and electrical conductivity. Moreover, it is planned to extend this technique to other electrically conductive adhesives (ECAs) to improve their thermal conductivity.

## 2. Experiment

### 2.1. Materials

In our research, the filler Ag powder (6 μm, thickness of 200–400 nm, flake) was obtained from Xinshengfeng Inc., Al_2_O_3_ powder (1 μm, sphere) was obtained from Baitu Inc., and epoxy resin (bisphenol A epoxy resin E-51) was supplied by Macklin Inc. Ancamine 1618, used as a curing agent, was purchased from Air Products and Chemicals Inc., and 3-Aminopropyltriethoxysilane, the coupling agent, was obtained from Meryer Chemical Technology Co., Ltd. In addition, the 2-Methylimidazole, obtained from Macklin Inc., and Allyl glycidyl ether, obtained from Runxiang Inc., were used as catalyst and diluent respectively.

### 2.2. Preparation of the Silver Epoxy Adhesive

**Figure 1:**
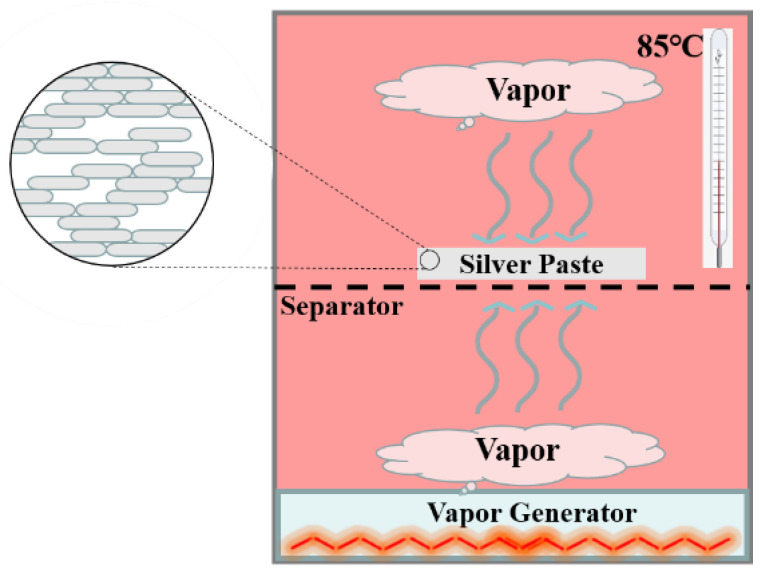
The schematic of treating the silver epoxy adhesive with vapor.

To prepare the silver epoxy adhesive, a mixture of epoxy resin, curing agent, coupling agent, catalyst, and diluent was created in the proportion of 100:100:4:2:20. Ag powder (85 wt%) was added to the mixture and thoroughly mixed. The resulting adhesive was further mixed using a homogenizer at a speed of 2000 r/min for 5 min. To enhance its curing performance, the adhesive was cured in a vacuum drying oven at 80 °C for 40 min and then at 120 °C for 20 min. This adhesive was labeled Ag-25, and had a diameter of 50 mm and a thickness of 1 mm. To investigate the effect of vapor treatment on the adhesive, Ag-25 was subjected to vapor in the atmosphere at a temperature of 85 °C for 24 h, and the resulting adhesive was named Ag-85-Vapor (see Figure 1). Ag-25 was also treated at a temperature of 85 °C without vapor as a control group, and this adhesive was labeled Ag-85.

### 2.3. Characterization

The fracture surface was analyzed using a scanning electron microscope (SEM, SU8230, HITACHI, Ltd., Tokyo, Japan). The composition was determined through X-ray diffraction (XRD; D8 Advance, Bruker, Germany). The content of H2O was measured using differential scanning calorimetry (DSC, METTLER TOLEDO, Ltd., Bussigny, Switzerland) and thermal gravimetric analysis (Discovery TGA 550, TA Instruments, Ltd., New Castle, DE, USA). Contact angle tests were performed using the DSA25 instrument (KRUSS, Ltd., Hamburg, Germany), and the porosity tests were conducted using the AutoPore V9620 instrument (McMuriartic, Ltd., Norcross, GA, USA). The thermal conductivity of the cured adhesive was tested using the LW-9389 tester (LONG WIN SCIENCE & TECHNOLOGY Co., Ltd., Shandong, China) with two copper heating plates each measuring 1 inch square. The electrical conductivity was tested using a Four-probe Tester (ST-2258C, Suzhou jingge Electronics Co. Ltd., Suzhou, China). This technique is commonly used in industry for measuring the volume resistivity of samples.

## 3. Results and Discussion

**Figure 2:**
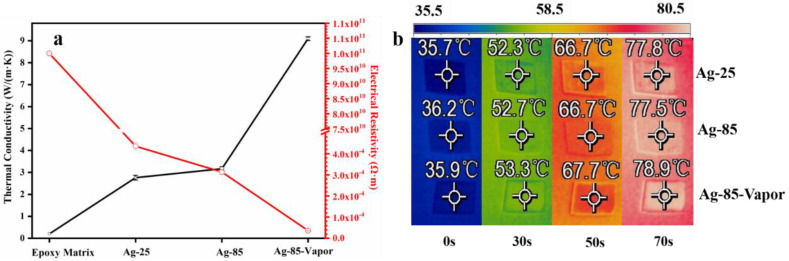
The property of silver epoxy adhesives (Epoxy matrix, Ag−25, Ag−85, and Ag−85-Vapor). (**a**) The thermal conductivity and electrical conductivity of silver epoxy adhesives; (**b**) the infrared images of the silver epoxy adhesive.

Figure 2 presents the results of thermal conductivity and electrical conductivity tests conducted on silver epoxy adhesive after three different curing treatments. As shown in Figure 2a, the thermal conductivity of Ag-25 and Ag-85 is similar. After treatment with the water vapor, the Ag-85-Vapor exhibits excellent thermal conductivity, reaching 9.1 W/(m·K), which is much higher than that of Ag-25 and Ag-85. This value is higher than the thermal conductivity (3–7 W/(m·K)) reported in the literature for a similar amount of silver powder [1,21,22]. Furthermore, the resistivity of Ag-85-Vapor is as low as 3.65 × 10^−5^ Ω·m, indicating significantly improved electrical conductivity. The improved thermal conductivity of Ag-85-Vapor is also confirmed by infrared thermography, as shown in Figure 2b.

**Figure 3:**
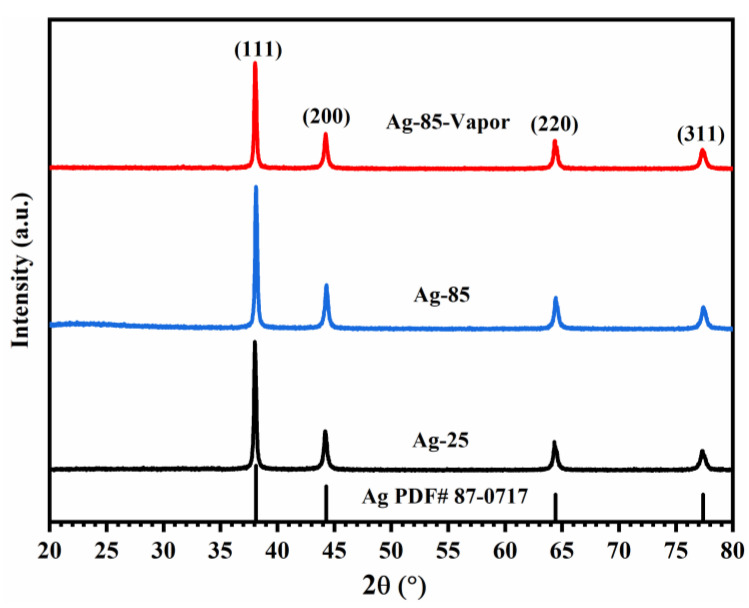
The XRD patterns of the silver epoxy adhesives (Ag-25, Ag-85, and Ag-85-Vapor).

Comparing the XRD analysis of Ag-25, Ag-85, and Ag-85-Vapor, as depicted in Figure 3, it is evident that all three samples exhibit the same phase composition, which is silver powder, without any impurities. Despite undergoing vapor treatment, no new phase is observed in Ag-85-Vapor, and the crystallinity of silver powder remains largely unchanged. Therefore, it can be inferred that the remarkable improvement in thermal conductivity and electrical conductivity of Ag-85-Vapor cannot be attributed to the formation of a new phase or the increase in crystallinity of silver powder during high-temperature treatment.

**Figure 4:**
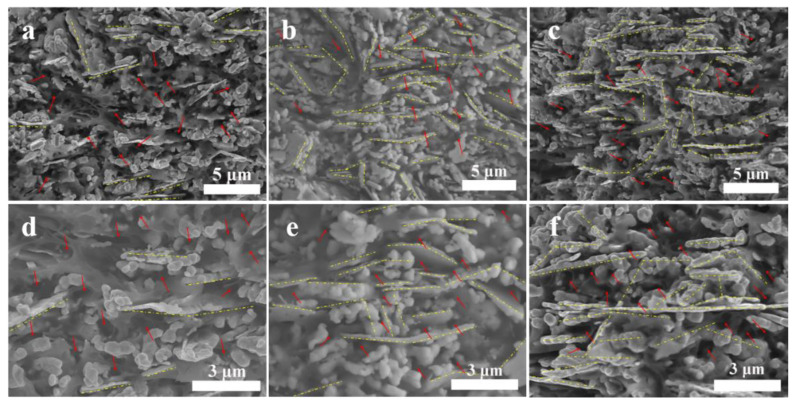
The SEM morphology of the silver epoxy adhesives. (**a**,**d**) Ag-25; (**b**,**e**) Ag-85; (**c**,**f**) Ag-85-Vapor.

Figure 4 illustrates the SEM morphology of Ag-25, Ag-85, and Ag-85-Vapor samples. It is evident from Figure 4a,d that, in Ag-25, the silver powder sheets are not closely overlapped and there are numerous pores between them, leading to relatively low thermal conductivity and electrical conductivity, as shown in Figure 2. In contrast, after undergoing high-temperature treatment, more sheets are overlapped in Ag-85 (Figure 4b,e) and Ag-85-Vapor (Figure 4c,f), forming more heat conduction paths. The increase in the number of paths facilitates the transmission of more electrons and phonons, resulting in a further improvement in thermal and electrical conductivity. Additionally, some voids are still present in Ag-85 and Ag-85-Vapor samples. Therefore, the filling of H_2_O between the voids may be responsible for the noticeable difference in thermal conductivity and resistivity.

**Figure 5:**
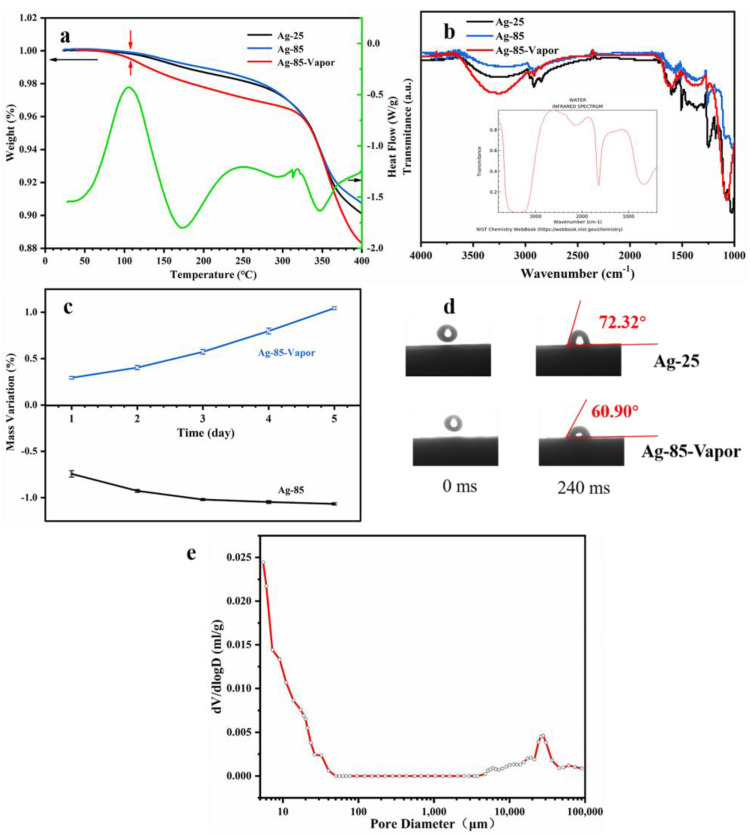
The characterization of the existence of H_2_O by treatment with vapor. (**a**) TG−DSC; (**b**) infrared spectrum; (**c**) weight variation with the treating time; (**d**) wettability test; (**e**) porosity test of Ag−85.

To confirm the presence of H_2_O after the vapor treatment, the study utilized TG-DSC and infrared spectroscopy for analysis. The TG-DSC results (Figure 5a) showed that free water would evaporate before 110 °C and that more H_2_O volatilized in Ag-85-Vapor. Comparing the results showed that under high-temperature vapor treatment, approximately 1 wt% of H_2_O entered the silver adhesive pores. According to the literature, the characteristic peaks of H_2_O in infrared absorption would be around 3300 cm^−1^ and 1600 cm^−1^ (Figure 5b) [23,24]. The infrared spectral analysis of Figure 5b indicated that Ag-85-Vapor had a stronger characteristic peak of free water, signifying the existence of more free water. The weight changes of Ag-85 and Ag-85-Vapor collected during the treatment indicated that 1–2 wt% H_2_O entered the silver adhesive pores during the treatment, which was consistent with the TG analysis results. The wettability test showed that the prepared epoxy silver adhesive had better hydrophilicity, with Ag-85-Vapor exhibiting a smaller wetting angle than Ag-85, indicating better hydrophilicity and easier access of H_2_O into the silver adhesive pores. Additionally, Figure 5e reveals that there were nano-micropores present in the Ag-85, and that H_2_O could enter these micropores through gross capillary action and remain there for a significant amount of time.

Figure 5 shows that during the vapor treatment process, H_2_O can penetrate into the gaps between silver sheets and significantly enhance the thermal and electrical conductivity of silver glue. To confirm this, several control groups were tested and their results are summarized in Table 1. The results of groups 1, 2, and 3 indicate that the coupling agent has no direct influence on the change in thermal conductivity before and after vapor treatment. On the other hand, group 4 shows that vapor treatment can effectively improve the thermal conductivity of silver epoxy adhesive, while the thermal conductivity of composite adhesive filled with Al_2_O_3_ is minimally affected. The superior thermal and electrical conductivity of silver epoxy adhesive at low filler concentration can be attributed to the excellent electron conductivity of Ag. The H_2_O existing between the sheets and voids allows electrons to pass through, thus improving the conductivity of the composite material. In contrast, Al_2_O_3_ is a covalent compound that is not conducive to electron transmission, which means that the vapor in the gap cannot act as a bridge for electronic conduction, resulting in insignificant improvement in thermal conductivity of its composite material [25]. These findings further confirm that H_2_O plays a crucial role in improving the thermal and electrical conductivity of silver epoxy adhesive, acting as a bridge for electronic conduction.

**Figure 6:**
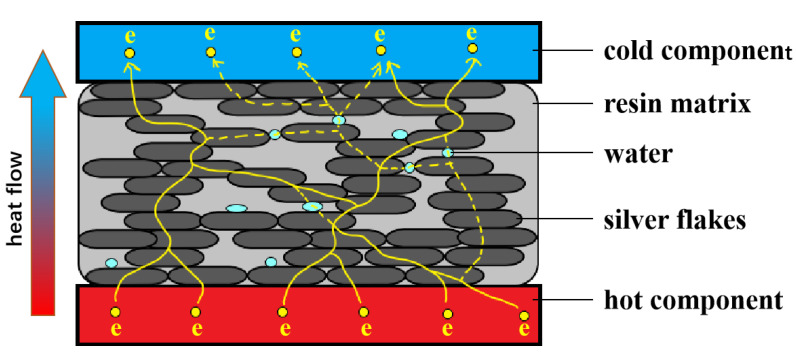
The schematic diagram showing how the treatment of vapor improves the thermal conductivity of the silver epoxy adhesive.

Based on previous research and analysis, we inferred the mechanism behind the improvement in thermal conductivity in silver epoxy adhesive due to vapor treatment, as depicted in Figure 6. Traditional curing methods result in the formation of pores in the adhesive, which obstruct the conduction of phonons and electrons. Consequently, the thermal and electrical conductivity of Ag-25 is low. However, vapor treatment at 85℃ causes the sheets of silver epoxy adhesive to overlap more closely due to high-temperature curing, thereby constructing more paths for heat conduction and promoting an increase in thermal conductivity.

Moreover, the silver adhesive has good hydrophilicity, and under vapor treatment, H_2_O vapor can enter the pores and lamella of the silver adhesive. As free water is an excellent conductor of electrons, electrons can travel through the traces of free water in the silver adhesive. Therefore, additional electron conduction pathways are constructed inside the silver epoxy adhesive, significantly improving its thermal and electrical conductivity.

## 4. Conclusions

In this paper, a research study on a simple and effective method to enhance the thermal conductivity of silver epoxy adhesive by treating it with water vapor is presented. The method leads to a significant improvement in the thermal conductivity of the adhesive, with a value of 9.1 W/(m·K), which is three times higher than the value obtained using traditional curing methods (2.7 W/(m·K)). The research revealed that the H_2_O present in the adhesive can penetrate the gaps and holes, creating additional paths for electron conduction, which significantly improves the thermal and electrical conductivity of the adhesive. Furthermore, the method is anticipated to improve the performance of other packaging materials and fulfill the high-performance requirements of ECAs.

## Figures and Tables

**Table 1:** The thermal conductivity of epoxy adhesive containing different fillers and coupling agents, treated with air and vapor at 85 °C, respectively.

NO	Filler	Coupling Agent (wt%)	Filling Content (wt%)	Filling Content (vol%)	Thermal Conductivity (85 °C) (W/(m·K))	Thermal Conductivity (85 °C-Vapor) (W/(m·K))
1	Ag	0	85.7	40.67	3.32 ± 0.05	7.19 ± 0.04
2	Ag	1	86.4	42.09	3.32 ± 0.03	7.84 ± 0.03
3	Ag	3	86.8	42.93	3.19 ± 0.09	9.1 ± 0.08
4	Al_2_O_3_	0	86.7	69.09	1.55 ± 0.04	1.64 ± 0.06
5	Al_2_O_3_	1	87.2	70.02	1.56 ± 0.05	1.64 ± 0.07
6	Al_2_O_3_	3	87.5	70.59	1.55 ± 0.07	1.78 ± 0.05

## Data Availability

All data that support the findings of this study are included within the article.
